# Managing ocular allergy

**Published:** 2024-10-02

**Authors:** Anahita Kate, Sayan Basu, Victor Hu

**Affiliations:** 1 Shantilal Shanghvi Cornea Institute, L V Prasad Eye Institute, Vijayawada, Andhra Pradesh, India.; 2 Shantilal Shanghvi Cornea Institute; Centre for Ocular Regeneration (CORE); and Brien Holden Eye Research Centre (BHERC); L V Prasad Eye Institute, Hyderabad, Telangana, India.; 3 International Centre for Eye Health, London School of Hygiene and Tropical Medicine UK.

**Ocular allergy is debilitating and potentially blinding, and it most commonly affects children and young adults. Successful step-wise treatment can, however, be life-changing.**

Ocular allergy is more common in hot, dry and dusty regions. It can particularly affect children and young people, causing social withdrawal and loss of schooling. Eye rubbing due to allergy can lead to keratoconus, so controlling the condition is important to prevent long term complications such as vision loss. In this article, we suggest a pragmatic management approach based on the severity of the condition. For more detailed information on the classification and management of ocular allergy, see the previous *Community Eye Health Journal* articles in the panel.^1,2^

**Useful articles from the *Community Eye Health Journal***

Bore M. Managing ocular allergy in resource- poor settings. *Community Eye Health*. 2016;29(95):47-49. https://bit.ly/3WIe43N

**Figure:**
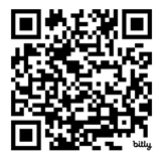

Rathi VM, Murthy SI. Allergic conjunctivitis. *Community Eye Health*. 2017;30(99):S7-S10. https://bit.ly/4ciwiP7

**Figure:**
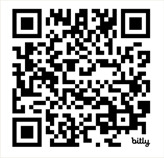

## Features and types of ocular allergy

Ocular allergy is a broad term that encompasses different types of allergic inflammation of the conjunctiva and/or cornea in response to allergens and irritants in the patient's environment.

It is usually **bilateral**, with symptoms and signs varying according to the patient and the type of ocular allergy.

The distinctive feature of ocular allergy is **intense itching** of the eyes. Patients may also experience burning sensation, sensitivity to light, and irritation.

**More mild** forms of ocular allergy affect the conjunctiva only. Patients with mild ocular allergy usually have one of the following two conditions:

- **Seasonal allergic conjunctivitis.** This usually occurs during the summer months, when pollen and other allergens are released.
- **Perennial allergic conjunctivitis.** This occurs throughout the year and may be a response to house dust mites, animal dander, or feathers.

**Figure 1:**
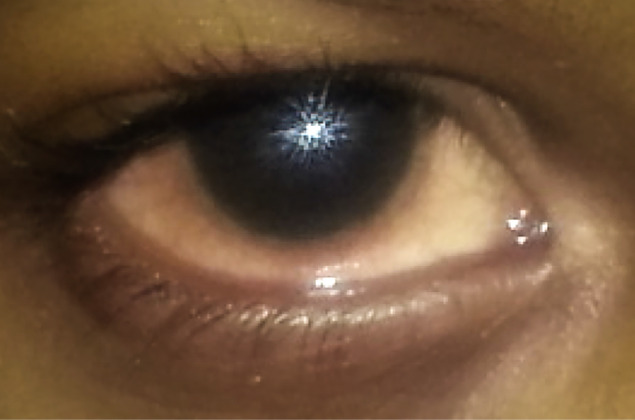
Red eyes (hyperaemia and limbal pigmentation)

**Figure 2:**
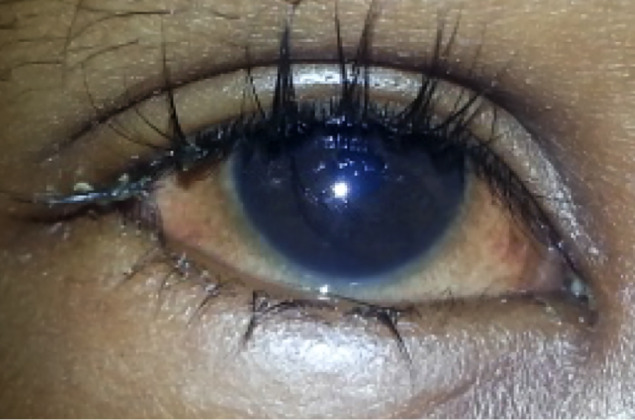
Mild, blister-like swelling of the conjunctiva (chemosis)

**Figure 3:**
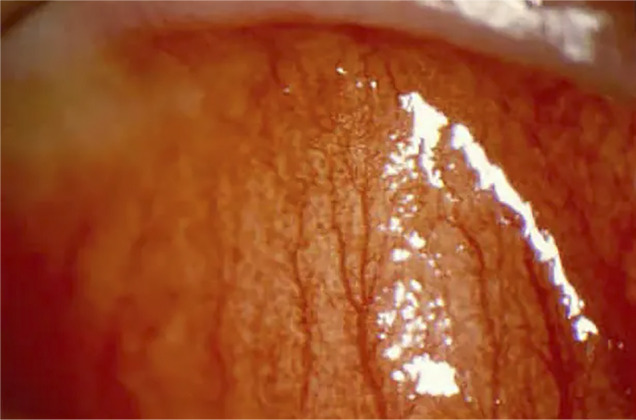
Slit lamp image showing small papillae in the upper eyelid. This patient has perennial allergic conjunctivitis.

**Signs of mild ocular allergy** include:

- Watering
- Red eyes (hyperaemia, see Figure 1)
- Mild blister-like swelling of the conjunctiva (chemosis, Figure 2)
- Small papillae inside the eyelids (less than 0.3 mm in diameter, see Figure 3)

**Signs of mild ocular allergy**

© MILLICENT BORE CC BY-NC-SA 4.0

© MILLICENT BORE CC BY-NC-SA 4.0

© ESEN AKPEK CC BY-NC-SA 4.0

Ocular allergy is considered **more severe** when there is corneal involvement. This can be the result of seasonal or perennial allergic conjunctivitis that has not been managed well, or if the patient has one of the following conditions:

- **Vernal keratoconjunctivitis.** This typically affects young males and tends to be worse in the spring/summer months.
- **Atopic keratoconjunctivitis.** This is more common in adults and is associated with eczema on the eyelid skin.

**Figure 4:**
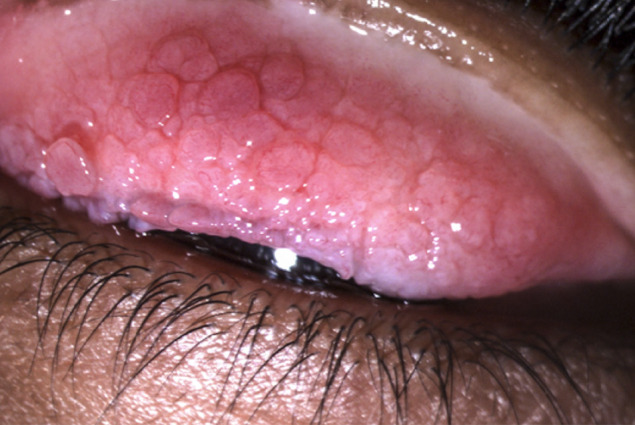
Large papillae, with a typical ‘cobble-stone’ appearance, in a patient with vernal keratoconjunctivitis.

**Figure 5:**
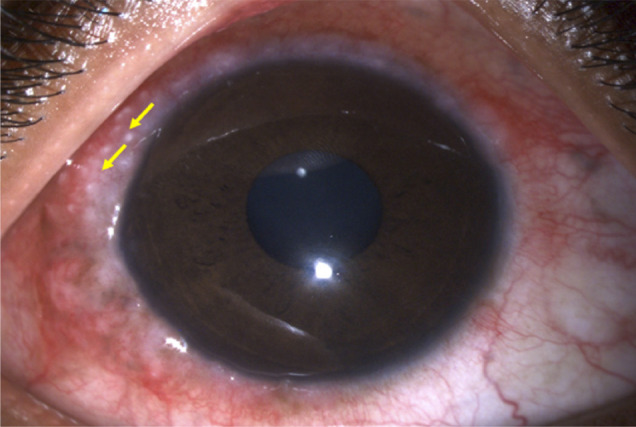
Limbal thickening due to inflammation. The small inflammatory nodules are known as Horner-Trantas dots.

**Figure 6:**
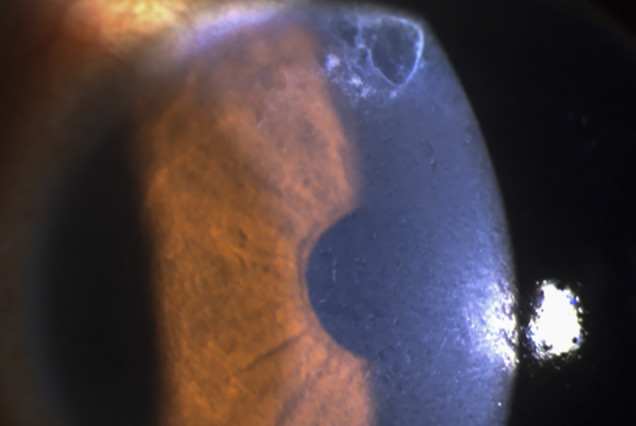
Shield ulcer in the upper part of the cornea.

**Signs of more severe ocular allergy** include:

- **Large papillae** (cobblestones) inside the eyelids (Figure 4); anything bigger than 0.3 mm is considered severe.
- **Limbal thickening** or inflammation, including small nodules known as Horner-Trantas dots (Figure 5).
- **Shield ulcer**. Papillae inside the eyelids can damage the surface of the cornea, eventually leading to the formation of a shield ulcer (Figure 6).

**Signs of more severe ocular allergy**

© V RATHI/LVPEI CC BY-NC-SA 4.0

© V RATHI/LVPEI CC BY-NC-SA 4.0

© V RATHI/LVPEI CC BY-NC-SA 4.0

**Note:** Any type of ocular allergy can result in sight loss if not managed well. Patients can develop keratoconus, or exacerbate it, if they excessively rub their eyes; advise all patients with ocular allergy to avoid eye rubbing and monitor them for signs of keratoconus (see articles in this issue). It is important to refer patients with more severe forms of ocular allergy, as well as patients with mild forms that do not respond to treatment, so they can be managed by an ophthalmologist.

## Managing patients with mild ocular allergy

Mild ocular allergies can often be managed in the community or at primary care level, by following steps 1–4 as set out below. However, if there is no improvement with treatment, any suspicion of corneal involvement, or any other signs of severe ocular allergy, refer your patient to a specialist eye care unit.

## Step1. Supportive therapy and patient education

Educating patients and their caregivers is a vital component of the management of ocular allergy.

**Avoid allergens, both confirmed and suspected**. This may be obvious, such as avoiding certain animals, including cats, or certain grasses. More frequent washing of pillowcases or bedding may also help. Testing for specific allergens can also be done where available (either by skin prick testing or blood testing), usually in an allergy clinic.

**Avoid eye rubbing.** This is critical, as rubbing sets off a vicious cycle of inflammation and further itching. It can also cause sight-threatening complications such as corneal infections and keratoconus. Cold compresses can help to relieve itching. These may be commercially available and are stored in the fridge or can be made by putting a clean face cloth into cold water, wringing out the excess water, and placing the cold compress on the closed eyes for 5–10 minutes. Alternatively, the face cloth can be wrapped around ice placed in a clean plastic bag.

**Use artificial tears.** This can help flush out allergens and inflammatory mediators and help soothe the eyes. Preservative-free formulations should be used. If patients are receiving multiple drops for severe allergy treatment (see step 3) artificial tears may be omitted to simplify treatment and to reduce costs.

**Never self-medicate using over-the-counter steroid drops.** If used inappropriately, or if their use is not monitored, these can have serious complications including corneal infections, glaucoma, and cataract.

## Step 2. Oral antihistamines

These are often available over the counter and can be helpful, especially in more mild disease such as seasonal allergic conjunctivitis and/or when there is associated allergic disease, such as eczema or rhinitis. They generally have few side effects and can be used long term, although some antihistamines are known to cause drowsiness and should be used with care.

## Step 3. Topical anti-allergy medications

Anti-allergy eye drops include antihistamines and mast cell stabilisers, and are also available as a combined formulation known as dual-acting agents.

**Antihistamines** have a very rapid onset of action; however, the duration of effect is very short. Hence, these medications are rarely used in isolation. Conversely, **mast cell stabilisers** can take 2–6 weeks to start working, yet they offer sustained benefits over time due to their membrane-stabilising effects.^1,2^ **Dual-acting agents** combine the properties of both agents, providing rapid onset and prolonged duration, and are generally preferred when available. Several molecules exist under each category and various meta-analyses have shown that the clinical efficacy of each drug is comparable to the other.^3^ These drops are generally safe, well tolerated, and can be used in the long term. As with other eye drops, preservative-free formulations are preferred.

## Managing patients with severe ocular allergy

### When to refer

Refer patients for specialist care if:

1. There is no improvement after the treatment recommended in steps 1–3
2. There is any corneal involvement
3. There are signs of more severe disease, such as large (cobblestone) papillae or persistent limbal inflammation.

These patients are likely to need further treatment (see steps 4–8).

### Managing ocular allergy in the eye department

In a specialist care setting, continue providing the treatment detailed in steps 1–3. Beginning at Step 4, work through the steps until the disease process is controlled. Treatment can then be cautiously stepped down.

**Note:** Adherence with treatment is particularly important for patients who have more severe forms of ocular allergy. Explain to patients and their caregivers that their condition needs long-term treatment, and that they should continue their treatment even if their eyes are feeling better. See our previous issue on ‘Medicines for eye health’ for useful guidance on adherence (https://www.cehjournal.org/medicines/).

## Step 4. Topical corticosteroids

Steroid eye drops are rapid and effective in treating ocular allergy. However, due to their side effects, their use needs to be limited.

Steroids are used frequently at the start of treatment (e.g., 4–8 times a day); they are then rapidly tapered (e.g., over 4–6 weeks) to bring the disease under control.

Some steroid drops appear to have less severe side-effects (e.g., on intraocular pressure) and are generally preferred. These include loteprednol, hydrocortisone, and fluorometholone drops. Within this group, loteprednol is recommended for eyes requiring repeated doses. This is because it is only active on the corneal surface, where it is needed. Because it is unable to enter the eye, complications such as cataract and glaucoma are less common.^6^

## Step 5. Topical calcineurin inhibitors

Cyclosporine and tacrolimus can be used as topical immunosuppressants in the eye and are useful as a long-term therapy. Because it takes a few months for them to take full effect, steroid drops are often started at the same time, as a short-term measure. Several studies have shown the efficacy of cyclosporine and tacrolimus in reducing the signs and symptoms of ocular allergy. Limited literature exists comparing these medications directly, but available studies have demonstrated no discernible difference in outcomes between them.^4,5^ Cyclosporin eye drops have been licensed in Europe and the USA for the treatment of allergic eye disease. Tacrolimus ointment, formulated for ocular use, is also available several countries; its once-nightly dosage is easier to use and can improve compliance among paediatric patients.

## Step 6. Supratarsal steroid injection

This surgical intervention is indicated in patients with severe papillary conjunctivitis with corneal changes, such as a shield ulcer. A long-acting steroid such as triamcinolone (0.5 ml of a 40 mg/ml preparation) is generally preferred, although a shorter acting steroid such as dexamethasone (0.5 ml of a 4 mg/ml preparation) can be used. The upper lid is everted and a cotton bud soaked in local anaesthetic used to numb the area around the upper tarsal border. The cotton bud can be used to lift the everted lid away from the globe. The steroid is injected with a 30 gauge (or similar) needle, just above the upper tarsal border. Children may require general anaesthesia, but with careful counselling, and establishing a good rapport, even young children can tolerate this procedure. The intraocular pressure should be monitored in case of a pressure rise, but this does not seem to be common. Although supratarsal steroids can effectively control the allergy, the effect is usually temporary and relapse of the disease may be noted after 4–6 months.^7^ Close monitoring is needed, and systemic immunosuppression may offer a better alternative.

## Step 7. Systemic immunosuppression

Where the above measures have been tried and the inflammation is not controlled, then systemic treatment to reduce the immune response may be required (where available), especially if there is corneal involvement and a risk of vision loss. This would normally involve a multidisciplinary team approach, in partnership with other specialties who are experienced with immunosuppression, such as immunologists or rheumatologists. A careful systemic evaluation is needed, as well as frequent monitoring after treatment has been started. There is little evidence or widely accepted guidelines on the choice of agent or duration of therapy. However, cyclosporine is commonly used, and discontinuation is considered if no relapses occur after 6–12 months.

## Step 8. Surgical intervention

A shield ulcer may be seen in severe ocular allergy, typically vernal keratoconjunctivitis (Figure 3). This usually develops on the superior cornea and an inflammatory infiltrate can develop in the base of the ulcer, requiring surgical debridement. The severe limbal variant of vernal keratoconjuntivitis can result in limbal stem cell deficiency. Patients with limbal stem cell deficiency with sparing of the visual axis are managed with systemic immunosuppression and scleral contact lenses.^8,9^ Limbal stem cell transplantation is required in patients with severe limbal stem cell deficiency involving the visual access.^8,9^

## Newer therapy modalities

Numerous emerging avenues of treatment are under investigation for managing ocular allergies. One of the most promising avenues is **allergen-based therapy**, which involves identifying the specific allergen to which the patient is sensitive and implementing tailored desensitisation therapy.^10^ **Conjunctival immunotherapy**, which targets the condition directly at the affected site,^11^ may help prevent systemic complications associated with these treatment modalities. The use of **anti-IgE monoclonal antibodies** and **interleukin inhibitors** may provide additional steroid-sparing alternatives.^1,12^ Future studies exploring the role of these novel therapeutic options will hopefully help to expand the armamentarium of ocular allergy management.
